# Printed Circuit Board Sample Expansion and Automatic Defect Detection Based on Diffusion Models and ConvNeXt

**DOI:** 10.3390/mi16030261

**Published:** 2025-02-26

**Authors:** Youzhi Xu, Hao Wu, Yulong Liu, Xiaoming Liu

**Affiliations:** School of Mechanical Engineering, Anhui University of Technology, Ma’anshan 243002, China; huoxula@163.com (Y.X.);

**Keywords:** PCB, defect detection, ControlNet, stable diffusion, ConvNeXt

## Abstract

Soldering of printed circuit board (PCB)-based surface-mounted assemblies is a critical process, and to enhance the accuracy of detecting their multi-targeted soldering defects, we propose an automated sample generation method that combines ControlNet and a Stable Diffusion Model. This method can expand the dataset by quickly obtaining sample images with high quality containing both defects and normal detection targets. Meanwhile, we propose the Cascade Mask R-CNN model with ConvNeXt as the backbone, which performs well in dealing with multi-target defect detection tasks. Unlike previous detection methods that can only detect a single component, it can detect all components in the region. The results of the experiment demonstrate that the detection accuracy of our proposed approach is significantly enhanced over the previous convolutional neural network model, with an increase of more than 10.5% in the mean accuracy precision (mAP) and 9.5% in the average recall (AR).

## 1. Introduction

Over recent years, the electronics and information sector, as the foundation and pillar of the digital economy, has been receiving unprecedented attention and development. Printed circuit boards (PCBs) play an indispensable role in the electronic information industry, and surface mount technology (SMT) on the basis of PCBs is also a crucial process. With the increasing degree of industrial automation, there is a need for higher precision and more reliable PCBs, but because of the unpredictable nature of reflow soldering in SMT, soldering can randomly generate defects that can influence the working capabilities of PCBs. Therefore, there is a need for PCB welding defect detection [1,2], as most of the current detection approaches require highly accurate segmentation as well as manual marking one-by-one extraction for detection, and the detection efficiency is not high. As deep learning research continues to develop, an increasing number of people will combine deep learning methods with defect detection. Although it can be very good to improve the detection efficiency, it requires a large number of datasets for training, and the number of existing datasets is generally insufficient, affecting the training effect of the deep learning model; at the same time, for the PCB, such a complex background of the target dataset defect detection effect is not particularly ideal.

For the purpose of solving the problem of the insufficient number of samples in the datasets, the more common method in the past was to first classify the dataset samples into two categories, defective samples and normal samples, and then use the adversarial generative network [3,4] to expand them. However, due to the unstable training of the adversarial network, it is easy to crash [5], and the PCB dataset sample images in this study contain both defective and normal detection targets, so it is impossible to expand the dataset using the adversarial network. In this paper, ControlNet [6] combined with the Stable Diffusion model [7] is utilised to expand this dataset, which can be used to control the generation of the image by extracting the traits from the input image along with adding a certain amount of noise, as well as combining it with the input cue words, thus obtaining images that are pixel-wise dissimilar to the input image.

Currently, the most commonly used methods in deep learning-based detection of targets are Faster R-CNN [8,9], Mask R-CNN [10,11], and Cascade R-CNN [12,13], which have obtained satisfactory outcomes for the field of defect detection. However, for datasets such as PCB soldering defect detection, which have complex backgrounds, multiple targets, and small differences between multiple targets, the detection accuracy of the above methods is not high enough to be applied to industrial production. We use the ConvNext network as the backbone network for image feature extraction, and the core idea of ConvNext is to use the components of the convolutional layer, fully connected layer, and pooling layer to extract image features [14]. The obtained features are fed into a feature pyramid network (FPN) [15] which performs a multiscale fusion of features to acquire a regular-sized image of the features. The Cascade Mask-RCNN network is then utilised as the detection head network for classification and detection.

In this research, we present this improved Cascade Mask-RCNN detection method using the ConvNext network, which can detect the soldering of all the surface assembled components in a single picture, improving the efficiency of PCB detection; the accuracy of the detection approach is also better than the original approach of detection.

The primary research findings of the article are the following:

1. Different from previous inspection methods that can only detect a single PCB soldered component, the proposed inspection method can detect defects in multiple soldered components within a single picture, which improves inspection efficiency.
2. Using the diffusion model to expand the PCB solder defect detection dataset with samples, the validity of the approach has been verified for the target detection results.
3. ConvNext is utilised as a backbone network to modify the Cascade Mask-RCNN detection algorithm to enhance the accuracy of classification and examination of the PCB soldering defect dataset.

The organisational structure of this article is as follows: In Section 2, we analyse the development of the study field of defect detection. Section 3 describes the theoretical knowledge of our research method. Section 4 carries out an experimental analysis and comparative experiments with previous methods. Section 5 summarises the whole paper.

## 2. Related Work

PCB defect detection has become a very critical technology in the modern electronics industry. Currently, the mainstream approaches to defect detection rely on machine learning approaches, which are mainly categorised into conventional machine learning approaches and deep learning-based approaches. As indicated in Table 1, the conventional machine learning defect detection method generally involves the extraction of features such as size, colour, and geometric shape of the target image, and then inputting these features into a classifier for classification. For example, Jiang [16] uses colour features for PCB solder paste defect detection, Xue [17] uses colour features for PCB defect point location detection, and Wu [18] uses random forests to classify defects based on the extraction of colour and geometric features of PCB solder joints and achieves high-accuracy experimental results. However, these traditional machine learning methods are time-consuming and labour-intensive to extract features in the case of complex samples and require the design of corresponding feature extraction algorithms for different detection tasks.

Deep learning detection algorithms for end-to-end analysis can be a good solution to the shortcomings of traditional machine learning approaches, but models for deep learning training need a great deal of defective datasets. In the past few years, researchers often used adversarial generative networks for image generation to expand the sample dataset. Zheng [19] and others combined convolutional neural networks and generative adversarial networks to create a deep convolutional adversarial network (DCGAN), which in turn achieves dataset expansion. Liu [20] uses the CycleGAN network to achieve LCD (Liquid Crystal Display) sample expansion and improve detection rates. All these adversarial generation network methods need to divide the samples into different two classes for competitive confrontation, i.e., defective and non-defective samples, but facing the PCB welding defects such as both defective and non-defective datasets does not achieve good results. The diffusion model is better than the GAN model (generative adversarial network) in image generation, and the stability is also good. Rombach et al. [7] proposed high-resolution image synthesis based on a potential diffusion model based on DDPMs (Denoising Diffusion Probabilistic Models) [21], which greatly improves the flexibility of the model. Zhang et al. [6] used ControlNet to control the inputs and outputs of the diffusion model, which makes the model very convenient to use. Peebles et al. [22] replaced the U-Net [23] network in the diffusion model with a Transformer [24] to improve the quality of sample generation. The diffusion model can be used for dataset sample expansion because it only needs to provide certain cues and original images to generate sample images that are highly similar to the original images.

Deep learning-based detection algorithms learn target image feature information by training neural networks, which can automatically recognise complex and variable target objects. Wang [25] used Faster R-CNN to detect defects on turbine blades, which improves the accuracy of defect detection and also verifies its superiority in detection speed. Wu [26] used Mask R-CNN to detect defects on circuit-board-resistor end soldering for defect detection, achieving classification detection and labelling of multiple defects. However, in the face of similar PCB electronic components, welding defects such as complex defect background, defect shape complex, and other cases, the above detection network does not achieve good results.

Therefore, in the face of PCB electronic component welding defect detection, due to the diversity of its detection target defects, the traditional machine learning methods are laborious and difficult, and the deep learning-based detection methods such as Faster R-CNN and Mask R-CNN do not have a high detection accuracy, so it is necessary to improve the above detection network. We utilise ConvNext as the backbone network to improve the Cascade Mask-RCNN detection algorithm to improve the accuracy of PCB soldering defect dataset classification and detection.

## 3. Proposed Method

The methodological framework of the experiment is to expand the PCB surface mount component dataset samples using ControlNet in combination with the Stable Diffusion Model, then use the expanded dataset to train our presented ConvNext Cascade Mask R-CNN model, and finally carry out the comparison experiments with other detection models.

### 3.1. Expansion of Data Samples Based on Diffusion Model

**Figure 1:**
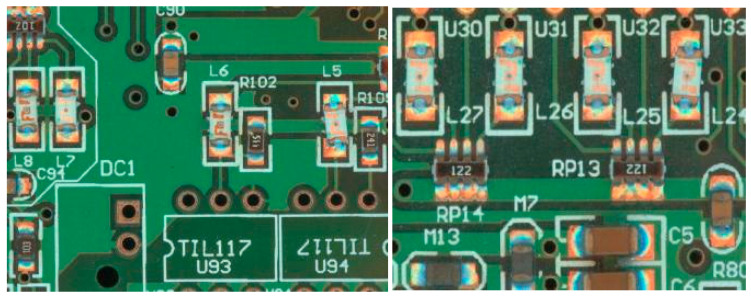
Soldering sample drawing for PCB surface assemblies.

**Figure 2:**
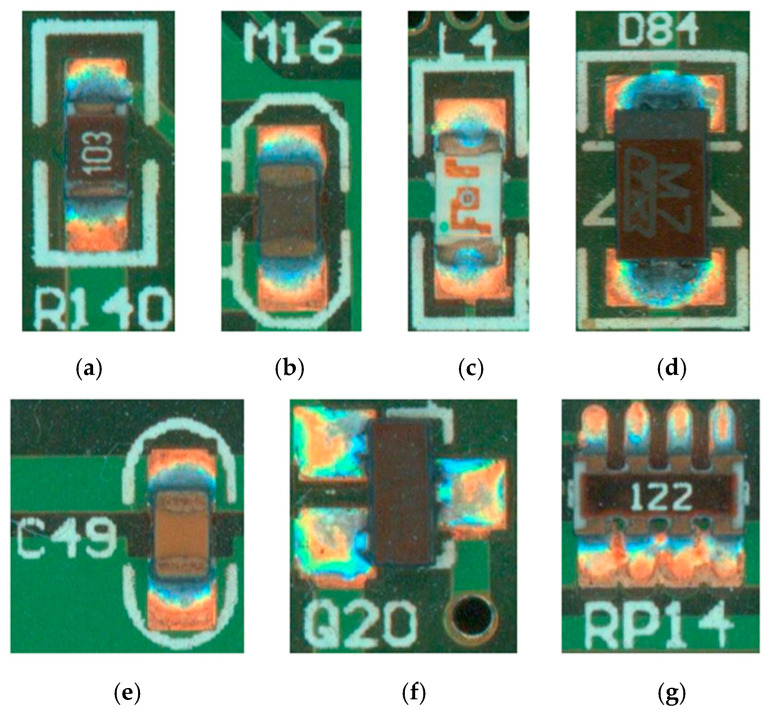
Objective installation component image: (**a**) resistor; (**b**) thermistor; (**c**) inductor; (**d**) diode; (**e**) capacitor; (**f**) triode; and (**g**) potentiometer.

The dataset used in this experiment is the surface-mounted component soldering dataset of PCB; as shown in Figure 1, the picture contains many medium-surface-mounted components, and the main objects of study in this paper are the resistor, capacitor, inductor, thermistor, diode, triode, and potentiometer, and their defect-free schematic diagrams are shown in Figure 2.

Since the PCB sample image contains multiple surface-mounted component targets, the detection target and background differences are not very large, and the PCB sample collection containing defective components is difficult, the original dataset needs to be expanded. This experiment uses a diffusion model-based approach for sample expansion. The initial attempt was to use the Stable Diffusion Model directly for generation. Firstly, the original sample pictures were fed in the pre-trained Stable Diffusion Model, encoded into the latent subspace by a VAE (Variational Autoencoder) [27], and then sampled by the DDIM (Denoising Diffusion Implicit Model) sampler. Meanwhile, the cue text was extracted as a vector input to the potential subspace by a CLIP (Continuous Liquid Interface Production technology) text encoder [28], which was combined with the feature images to further enhance the output of the control model. Finally, the potential features with added noise were sampled and decoded with VAE to obtain the target output image. However, since the output image still differed significantly from the initial image to a certain extent, it did not correspond to objective reality and could not be used for dataset expansion, so it needs to be improved.

**Figure 3:**
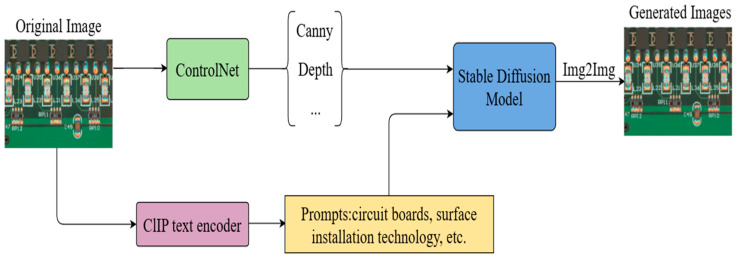
Sample generation model.

Introducing ControlNet to the Stable Diffusion Model enables us to increase our control over the output of the diffusion model. As shown in Figure 3, ControlNet can extract features such as Canny or Depth from the input image, combine them with textual information, and input them into the Stable Diffusion Model for iteration to generate pictures that are similar to the input samples.

### 3.2. Target Detection Based on R-CNN Series of Models

Because of the excellent performance of deep networks such as Alex-Net [29] and VGG [30] in classification detection tasks, people found that the features autonomously extracted by deep learning methods were better than those extracted manually by traditional methods, so they began to use deep learning methods in more detection tasks. At that time, target detection algorithms mainly used the colour, texture and other features of the image itself, and the R-CNN series of algorithms introduced CNN and a proposal for detection. Faster R-CNN largely consists of four modules: feature extraction network, RPN, ROI Pooling (Region of Interest Pooling), and classification and regression, which achieve a faster and more accurate detection of targets. The feature extraction network is composed of a deep convolutional network that is utilised for extracting the feature map of the picture, and the RPN is a region candidate network that is utilised for generating candidate regions efficiently and achieving end-to-end network training.

The Mask R-CNN model adds an extra Mask branch on top of the Faster R-CNN, which can be used to generate pixel-level masks. At the same time, the ROI Pooling in the Faster R-CNN model is substituted with ROI Align, which is used to deal with the problem of misalignment between the Mask and the target in the original image. In contrast to the Faster R-CNN model, it is able to perform semantic segmentation and objective detection simultaneously, which also improves the target detection performance.

The Cascade Mask R-CNN model is further improved on the basis of Mask R-CNN, which reduces the misdetection rate and improves the detection speed and accuracy by adopting a cascade structure. The cascade architecture is composed of several stages, the input of the former stage is the input of the latter stage, the quality of the input gradually becomes better, the target detection threshold gradually increases, and the quality of the output gradually becomes better, and at the same time, the misdetection rate can be significantly decreased. Therefore, compared with the original Mask R-CNN model, the increased cascade structure can better handle the multi-target detection task in some complex scenes.

The backbone networks of the above series of R-CNN models are adopted as VGG or ResNet networks, which are convolutional neural networks that rely on convolutional kernels of fixed size and pooling layers, which results in long-range feature information often being lost during feature sampling. The more convolutional layers there are, the more contour information about the object is lost. As a result, when detecting solder defects in PCB surface mount assemblies, it often is not possible to meticulously identify the appearance of the weld and different components simultaneously, and improvements to the backbone network are needed.

### 3.3. ConvNeXt Backbone

**Figure 4:**
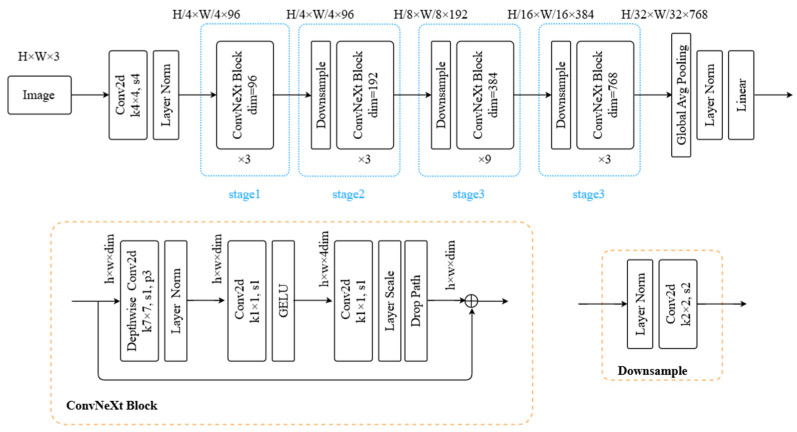
ConvNeXt backbone.

ConvNeXt is a novel architecture based on convolutional neural networks (CNNs), which aims to upgrade the traditional convolutional structure by combining the advantages of traditional convolutional networks and Visual Transformers to enhance the efficiency of visual detection missions without sacrificing the efficiency. The ConvNeXt model compares with traditional CNNs in terms of the convolutional layers; ConvNeXt samples a larger convolutional kernel and a deeper network structure to catch a more extensive range of image context information, which helps to raise the model’s comprehension of long-distance dependencies in the image. ConvNeXt also employs deeply separable convolution, which helps to reduce the model parameters and increase the computational efficiency. ConvNeXt employs a layer normalisation technique after each convolutional block instead of the batch normalisation of traditional CNNs, which helps to accelerate the convergence and stabilise the training process. To enhance the effectiveness of the model training process and its expressive power, ConvNeXt adopts a multilayer perceptron (MLP) structure similar to that of ViT, i.e., two fully connected layers sandwiched by a GELU activation function, and uses a deep hierarchical design to construct a multiscale representation of the visual features by incrementally downsampling and increasing the dimensionality of the features, which in turn allows the model to learn from low- to high-level visual traits. The schematic of this model is shown in Figure 4, where k stands for convolutional kernel, s stands for stride, and p stands for padding. Firstly, the input image is resized and normalised, feature extraction is performed using the four-stage ConvNeXt module, feature aggregation is performed by the Global Avg Pooling module, and finally, the results are categorised and outputted.

### 3.4. Defect Detection Method Based on ConvNext Cascade Mask R-CNN

In this article, the ConvNext model network is used as a backbone network to modify the conventional Cascade Mask R-CNN model to enhance the inspection performance of soldering flaws in PCB surface-mounted assemblies. By introducing the ConvNeXt model architecture, the combination of its large convolutional kernel and self-attention mechanism can fully utilise the spatial information, improve the feature expression ability, and effectively reduce the number of parameters and computational burden through the depth-separable convolution. Therefore, using ConvNeXt instead of ResNet as the backbone network can improve the detection performance of the model in complex backgrounds.

**Figure 5:**
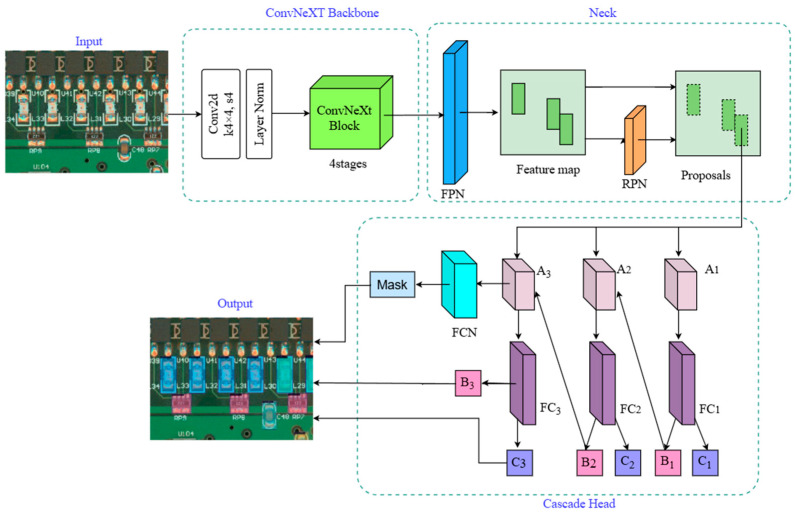
ConvNext Cascade Mask R-CNN-based defect detection model.

The network model of our proposal approach is presented in Figure 5. Pre-processing is completed by putting the input images through resizing and normalisation. After that, the feature extraction stage is carried out through the four-stage ConvNeXt module, which combines techniques such as depth-separable convolution, nonlinear activation, and normalisation to ensure that the model can efficiently learn and express features in complex tasks and obtain richer feature information. This is then followed by global average pooling to drastically reduce the feature dimensions and better catch the overall traits of the picture. The output feature information at different scales from each stage is fused using FPN. Next, a region suggestion network (RPN) [10] is utilised for producing and locating the detection frames so as to acquire proposals in the feature map. This is then fed into the Cascade Mask-RCNN of the head detection network. The Cascade Mask-RCNN consists of three stages of the perceptron. The first-stage perceptron takes the detection candidate frames output from the RPN as inputs and passes through the fully connected layer (FC) twice, while a relatively large detection threshold is set for the initial screening to acquire the preliminary classification outcome C1 and bounding box B1. The second-stage perceptron takes the outcome obtained in the first stage as input and sets a smaller detection threshold to improve the detection sensitivity, obtaining the second-stage result C2 and the optimised bounding box B2. The third-stage perceptron takes the results obtained in the second stage as input to obtain the final results C3 and B3. Afterwards, C3 and B3 are input into a four-layer fully convolutional network (FCN) [31] to generate the mask segmentation, and finally, the mask results are mapped back to the original image, making the detection results intuitively presented.

## 4. Experimental Results

### 4.1. Experimental Results of Data Sample Expansion on the Basis of Diffusion Model

The approach used in this experiment is to combine ControlNet with the Stable Diffusion Model. Firstly, suitable sample cue words need to be obtained. CLIP is used to reverse-derive sample cues from sample images, and some of the more frequent sample cues are considered assistant cues for the subsequent Img2Img. After a number of comparisons, the commonly used sample prompt words that we selected were circuit boards, surface mounting technology, electronic assemblies, motherboard wires, soldering, and so on.

**Figure 6:**
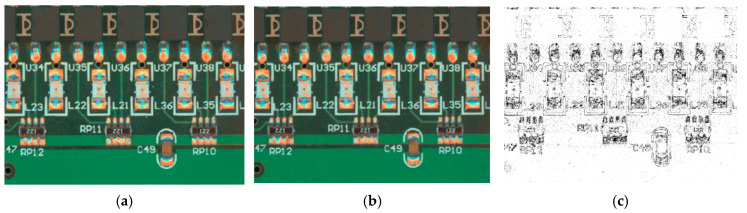
Sample generation results: (**a**) original sample image; (**b**) generated sample image; and (**c**) difference between the original sample image and the generated sample image.

We input the obtained cue words together with the original sample images to the Stable Diffusion Model and performed DDIM sampling using the open-source V1-5-Pruned-Emaonly.ckpt model, where the sampling paces are set to 20 steps. Afterwards, the model output was modulated using ControlNet, and the redraw intensity was set to 0.3. The original and generated sample images are represented in Figure 6, and Figure 6c represents the difference plots acquired by subtracting the original sample image from the generated sample image, which is then binarised.

Figure 6c illustrates that there is a non-negligible difference at the pixel level from the produced sample picture to the original sample picture, despite the fact that they are difficult to distinguish visually. This indicates that the produced sample image is based on objective reality, and the discrepancy at the pixel level will be transformed into a new tensor after being computed by the neural network. This not only enhances the robustness of the method but also effectively prevents overfitting situations. Next, the original sample pictures are commented on using the LabelMe (5.4.1) [32] labelling tool, and this label information is also adapted to the generated sample images, which can be combined to form a completely new training dataset for automatically expanding the training samples. Utilising this approach, we expanded the PCB surface-mount-assembly solder defect detection sample to 440, of which 200 images contained multiple inspection objects.

Table 2 below represents the experimental comparison results before and after sample expansion of the ControlNet-based PCB dataset; by comparing the experimental results, it can be found that the detection accuracy after expansion is significantly higher than the accuracy of the original unexpanded dataset, with an improvement of 0.038 (4.5%) on mAP_bbox_, 0.029 (3.1%) on mAP_bbox__50, 0.029 (3.1%) on mAP_bbox__75, and 0.027 (3.1%) on AR. The effectiveness of our adopted method can be proved.

### 4.2. ConvNext Cascade Mask R-CNN-Based Defect Detection Experiments

#### 4.2.1. Experimental Dataset and Parameter Settings

**Figure 7:**
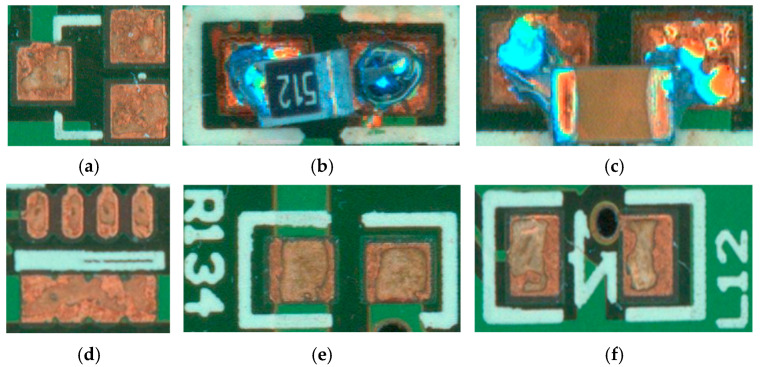
Example image of some of the defects: (**a**) missing triode; (**b**) tombstone resistor; (**c**) shift capacitor; (**d**) missing potentiometer; (**e**) missing resistor; and (**f**) missing inductor.

The target dataset of this experiment is the soldering defect dataset of PCB surface mount assembly, which is mainly derived from the actual production. This inspection contains seven inspection targets. According to the common types of defects in actual production, 19 labelling categories are set. They are normal, missing, offset, and monumental for resistors; normal, missing, offset, and monumental for capacitors; normal, missing, and offset for inductors; and normal and missing for diodes, transistors, thermistors, and voltage dividers. Figure 7 displays part of the defective species samples. Through preliminary experiments, we found that the samples were detected between 40,000 and 80,000 pixels with a pixel share of 6% to 12%. Therefore, when the thresholds for the three sensors in the cascade are selected to be 0.5, 0.6, and 0.7, in turn, high accuracy can be achieved while maintaining a small recall. The learning rate is in the setting of 0.0002, 200 epochs are trained, the training strategy is the simultaneous learning rate warm-up and decay, and the optimisation algorithm is AdamW.

#### 4.2.2. Comparison of Experimental Results of Defect Detection

The method of this experiment is the defect detection approach on the basis of ConvNext Cascade Mask R-CNN, which was carried out by comparing the Faster R-CNN, Mask R-CNN, Cascade Mask R-CNN, and ST–Mask R-CNN (Swin Transformer Mask-RCNN) defect detection methods to show the superiority of our methods in detecting solder defects in PCB surface-mounted assemblies, as shown in Table 3. After comparison of the mean accuracy (mAP) and average recall (AR) of the detection boxes under various IoUs, it is observed that our presented approach improves by 0.083 (10.5%) compared to the original network (Cascade Mask R-CNN) in mAP_bbox_, 0.04 (4.4%) in mAP_bbox__50, 0.044 (4.4%) in mAP_bbox__75, and 0.044 (4.8%) and AR by 0.078 (9.5%).

However, by comparing the number of Params and Flops, it can be seen that our proposed method is more complex than the original network (Cascade Mask R-CNN), its model parameters and Flops values are larger than those of the original network, and the computation takes more time cost.

**Figure 8:**
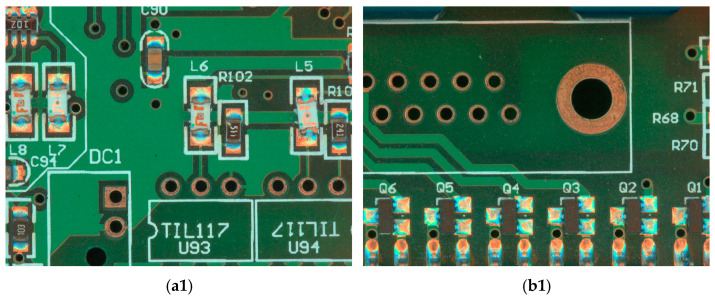

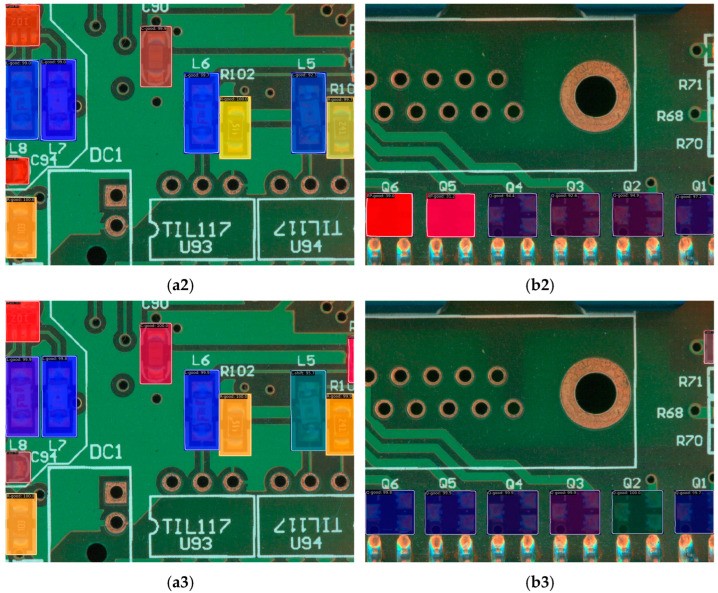
Schematic image of some test results: (**a1**,**b1**) original target detection image; (**a2**,**b2**) detection of resultant images using Cascade Mask R-CNN model; and (**a3**,**b3**) detection of resultant images using our proposed model.

In Figure 8, we display some model detection results, and by comparing our proposed method with Cascade Mask R-CNN, it is clearly found that the original Cascade Mask R-CNN network model is prone to errors and fails to achieve the expected results for multiple detection targets. For example, in (a2), it detects the inductor shift as normal inductance, and in (b2), some of the triodes are wrongly detected as other components. By contrast, our presented approach of the model with ConvNeXt as the backbone network ensures high accuracy.

## 5. Conclusions

For PCB surface component welding, which is a multi-target dataset containing both defects and normal detection, the common data sample expansion method based on the GAN model and the use of the Cascade Mask R-CNN detection network for target detection do not achieve good results.

Therefore, for dataset expansion, this paper proposes an automatic sample generation method that combines ControlNet and a Stable Diffusion Model. This method can quickly obtain high-quality sample images to expand the dataset. Meanwhile, this paper proposes the Cascade Mask R-CNN model with ConvNeXT as the backbone, which performs well in dealing with the multi-target detection task, and the detection accuracy is significantly improved, exceeding the former convolutional neural network model, with the average accuracy improved by more than 10.5% and AR improved by 9.5%. The proposed method in this paper has higher detection accuracy and enhances defect recognition in multi-target detection. However, its model is larger and requires higher computational resources. In the future, we hope that higher detection accuracy can be achieved with less computational resources, bringing higher production efficiency and more reliable product quality.

## Figures and Tables

**Table 1:** Classification of defect detection methods and their advantages and disadvantages.

Method Type	Name	Advantage	Disadvantage
Traditional Machine Learning Detection Methods	Classifier detection method based on image features	The method is simple and uncomplicated	Time-consuming and not very accurate
Deep Learning-Based Detection Algorithms	Faster R-CNN, Mask R-CNN, etc.	Target defects can be detected automatically	Poor classification and detection accuracy for multiple-target defective items
	Our proposed methodology	High accuracy of classification and detection of defective items with multiple targets	Larger model, longer computation time

**Table 2:** Comparison of experiments before and after dataset expansion.

	mAPbbox	mAPbbox_50	mAPbbox_75	AR
Original dataset	0.838	0.925	0.925	0.870
Expanded dataset	0.876	0.954	0.954	0.897

**Table 3:** Comparative experimental results of different models in PCB surface-mounted component dataset.

Model	mAP_bbox_	mAP_bbox__50	mAP_bbox__75	AR	Params	Flops
Faster R-CNN [9]	0.759	0.921	0.905	0.790	41.44M	0.178T
Mask R-CNN [11]	0.746	0.926	0.919	0.792	43.75M	0.258T
Cascade Mask R-CNN [12]	0.793	0.914	0.910	0.819	77.09M	0.390T
ST–Mask R-CNN [33]	0.853	0.937	0.934	0.872	47.37M	0.262T
Our proposed method	0.876	0.954	0.954	0.897	85.84M	0.472T

## Data Availability

The data that support the findings of this study are available from the corresponding author, H.W., upon reasonable request.
